# App-Based Hearing Screenings in Preschool Children With Different Types of Headphones: Diagnostic Study

**DOI:** 10.2196/44703

**Published:** 2023-11-03

**Authors:** Pornsek Tananuchittikul, Kwanchanok Yimtae, Nichtima Chayaopas, Panida Thanawirattananit, Pornthep Kasemsiri, Patorn Piromchai

**Affiliations:** 1 Department of Otolaryngology, Faculty of Medicine, Thammasat University, Pathumthani, Thailand; 2 Department of Otorhinolaryngology, Faculty of Medicine, Khon Kaen University, Khon Kaen, Thailand

**Keywords:** hearing screening, mobile application, mobile app, headphones, mobile health, mHealth, hearing, headphone, pediatric, child, preschool, audiology, audiometry, audiologist, screening

## Abstract

**Background:**

Hearing disability in preschool children can delay or impact oral communication and social skills. Provision of hearing screening tests by standard audiometry in low- to middle-income countries is problematic due to a lack of pediatric audiologists, standard hearing equipment, and standard soundproof rooms. Therefore, an innovative hearing screening tool that is easily accessible and inexpensive such as a mobile app should be considered. Headphones have been a crucial part of hearing screenings. Audiometric headphones, which serve as the reference standard, have been used in most studies. However, since audiometric headphones are not accessible in rural areas, we hypothesized that generic headphones can also be used in hearing screenings.

**Objective:**

This study aimed to determine the sensitivity, specificity, κ coefficiency, and time consumption of the PASS-Pro (Preschool Audiometry Screening System–Pro) app when using TDH39 headphones, Beyerdynamic DT 770 PRO headphones, and generic earmuff headphones compared to standard conditioned play audiometry.

**Methods:**

We recruited preschool children aged 4 to 5 years to participate in this study. The children received 3 PASS-Pro screening tests using different types of headphones in a quiet room and 1 standard conditioned play audiometry in a soundproof room. All tests were administered in random order. The agreement coefficient, sensitivity, specificity, and mean test duration were determined.

**Results:**

A total of 44 children participated in this study. For mild hearing loss screening, the κ coefficients between standard conditioned play audiometry and the PASS-Pro app using TDH39 headphones, Beyerdynamic DT 770 PRO headphones, and generic earmuff headphones were 0.195, 0.290, and 0.261 (*P*=.02, *P*=.002, and *P*=.004), respectively. The sensitivity for all headphones was 50% and the specificity was more than 88%. For moderate hearing loss screening, the κ coefficients were 0.206, 0.272, and 0.235 (all *P*s=.001), respectively. The sensitivity for all headphones was 100% and the specificity was more than 92%. There were no statistical differences in sensitivity and specificity between the reference headphone (TDH39), Beyerdynamic DT 770 PRO headphone, and generic earmuff headphones (all *P*s >.05). The PASS-Pro app used significantly less time to carry out hearing tests than conditioned play audiometry (*P*<.001).

**Conclusions:**

The PASS-Pro app, used with generic headphones, is effective for conducting hearing screening tests in preschool children with high sensitivity and specificity.

## Introduction

Hearing loss or hearing difficulties is a common cause of disability among people around the world. In 2012, the World Health Organization reported that 360 million people experienced hearing loss, indicating a prevalence of 5.3% in the global population (adults: 9%; children: 1%) [1]. In children aged less than 5 years, the prevalence of hearing loss was 2% (7.5 million) and the incidence of permanent hearing loss in preschool children was 3.4 to 3.56 per 1000 [2-5]. Early detection and intervention can help to improve patients’ quality of life [6].

In low- to middle-income countries, hearing difficulties affect people’s quality of life more than those living in high-income countries due to limited resources, such as medical staff, audiologists, hearing screening tools, and accessibility to medical treatment [7]. Hearing disabilities also affect preschool children in many ways, such as psychomotor skills, social skills, learning skills, emotional skills, and development skills [8,9]. Therefore, hearing screening tools play an important role in detecting hearing loss in preschool children and serve to prevent the disadvantageous outcomes described above.

Currently, standard hearing screening for children remains a major concern due to the lack of professional pediatric audiologists and soundproof rooms. The standard hearing test that is widely accepted for use among newborns to children aged 3 years is otoacoustic emission or automated auditory brainstem response and the test for children aged 3 to 6 years is the pure tone audiometry sweep test or conditioned play audiometry [10]. These tests require the cooperation of children, as well as professional audiologists, standard equipment, and standard soundproof rooms.

In our previous study [11], we tested the PASS (Preschool Audiometry Screening System) app with standard audiometric TDH39 headphones in 122 children aged 4 to 5 years. We found good overall sensitivity and specificity, indicating the potential of the app for use as a mobile hearing screening tool.

We designed the app for limited-source areas, rural areas, and areas without medical services. However, we found that audiometric TDH39 headphones were inaccessible in the rural area under study. In this study, we further evaluated the diagnostic value of the PASS-Pro app using 3 types of headphones as well as generic headphones. We selected the comparison headphones according to their availability in the Thai market and their low to moderate price range.

The objective of this study was to compare the sensitivity, specificity, κ coefficiency, and time consumption between standard conditioned play audiometry and the PASS-Pro (Preschool Audiometry Screening System–Pro) app using TDH39 headphones, Beyerdynamic DT 770 PRO headphones, and generic earmuff headphones.

## Methods

### Participants

Children in northeastern Thailand were recruited between November 2020 and February 2021. The inclusion criteria were as follows: children aged 4 to 5 years, who were able to communicate using the Thai language, and who were able to cooperate with the audiologists and medical staff. The exclusion criteria included pathology on the pinna and external auditory canal (which could interfere with headphone insertion), blurred or distorted vision, and blindness. The withdrawal criteria included being uncooperative during the study or incomplete hearing screening tests.

### Headphone Specifications

The specifications of the headphones used in this study are described in Table 1.

**Table 1.** Headphone specifications.

	TDH39 with earmuffs	Beyerdynamic DT 770 PRO	Generic headphones with earmuffs
Impedance	10 Ohms	32 Ohms	32 Ohms
Frequency response	100-8000 Hz	5-350,000 Hz	Up to 4 kHz with an approximate 10-dB attenuation from the maximum output
Sensitivity	108 dB SPL^a^/1 mW at 1 kHz	96 dB SPL	Estimated at 90 dB SPL
Total harmonic distortion	<1%	<0.2%	Approximately 10% at 1 kHz
Ambient noise isolation	30 dB	20 dB	30 dB

a SPL: sound pressure level.

### Study Flow

**Figure 1.**
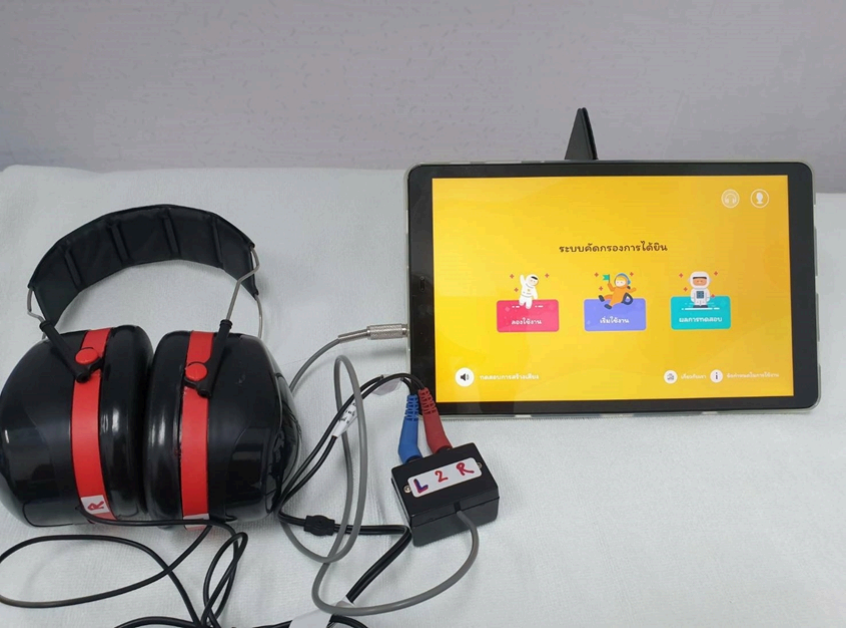
The PASS-Pro (Preschool Audiometry Screening System–Pro) app with TDH39 headphones.

All participants received otoscopic examination and hearing tests a total of 4 times: 3 PASS-Pro screening tests using different types of headphones (TDH39, Beyerdynamic DT 770 PRO, generic headphones with earmuffs) in a quiet room and 1 standard conditioned play audiometry in a soundproof room. All tests were randomly allocated to the participants (Figure 1).

An otolaryngologist examined each child’s external ears prior to the intervention. All audiologists in this study were blinded to the results of the hearing tests and the time spent doing each test to avoid bias.

### Test Protocol

**Figure 2.**
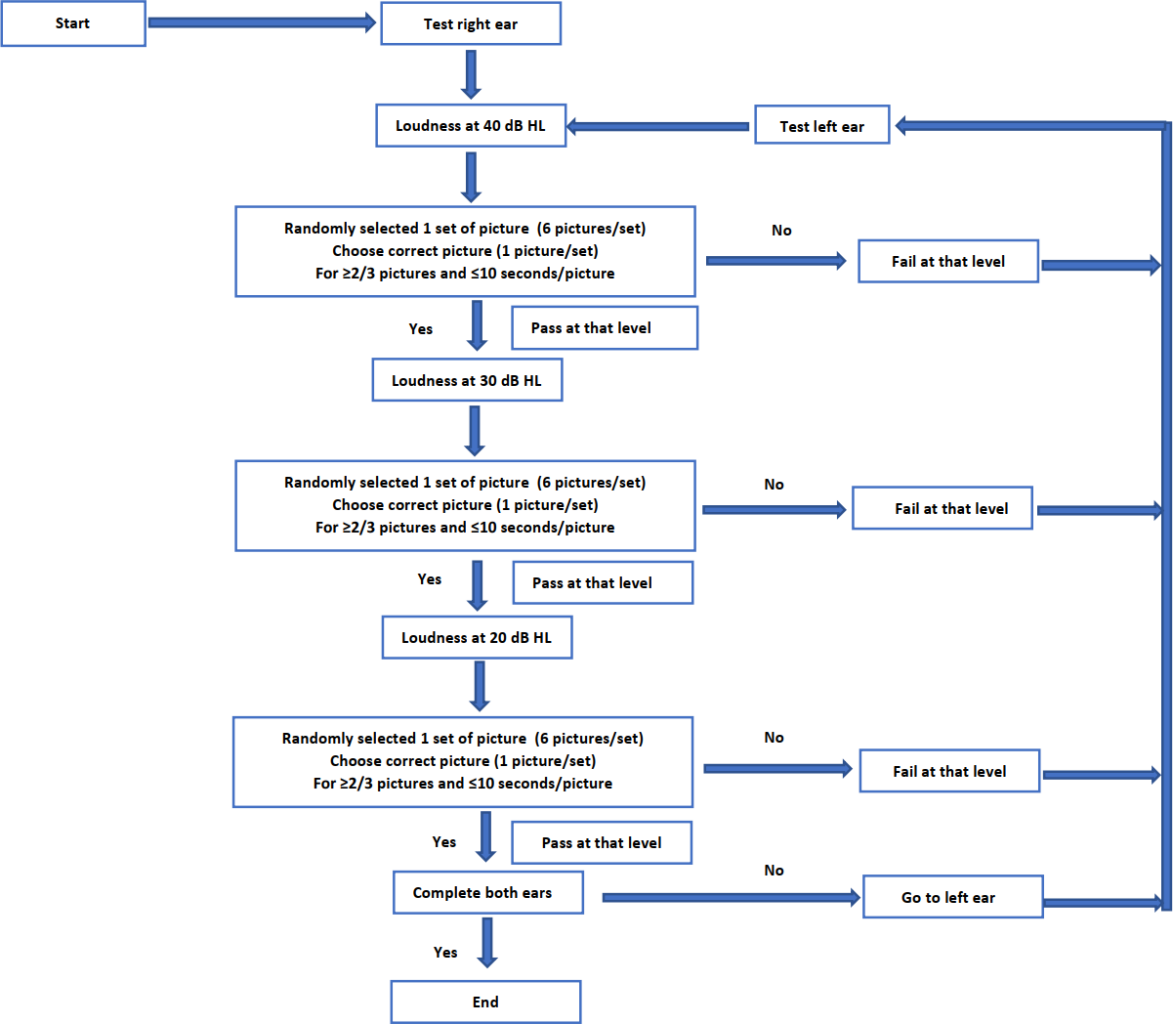
Test protocol. dB HL: dB hearing level.

The PASS-Pro app was accessed through the Samsung Galaxy Tab A 10.5 Android tablet. Starting with the right ear at 40 dB, the app randomly provided 1 set of pictures (6 pictures per set), then sounded a 2-syllable word that corresponded to one of the pictures. The participant had 10 seconds to choose the correct picture after hearing the word. The app recorded whether the answer was correct or not before the next random set of pictures were displayed. The participant had to select the correct picture again for each level of hearing as 3 new sets of pictures were presented. Obtaining 2 out of 3 correct responses meant the participant had passed the hearing level. Subsequently, the app reduced the volume from 40 dB to 30 dB, then to 20 dB. The process was repeated with the left ear (Figure 2).

The app stopped if the participant chose the correct picture fewer than 2 out of 3 sets of pictures in the same hearing level or did not choose the picture within 10 seconds. This was reported as hearing test failure for that level. The test results showed the hearing threshold and the time taken for each ear.

### Reference Standard

The children’s hearing threshold was evaluated using air-conduction pure tone audiometry at 0.5, 1, 2, 3, and 4 kHz. Participants with hearing loss, confirmed by the audiometry, will be sent to the otolaryngologist for standard evaluation and treatment.

### Test Environment

Hearing screening with the PASS-Pro app was conducted in a standard quiet room at a hospital. The pure tone audiometry was conducted in a soundproof room.

### Hearing Level Definition

According to the American Academy of Audiology’s childhood hearing screening guidelines [10], a hearing level of ≤20 dB was defined as normal. A hearing level between >20 dB and ≤40 dB was defined as mild hearing loss and a hearing level of >40 dB was defined as moderate hearing loss [10].

### Sound Equalization, Speech Signal Measurement, and Calibration

To calibrate the system to prepare for the trial, we first equalized the word files to have an equal root mean square. We then calibrated each word individually at 20, 30, and 40 decibel hearing level (dB HL) using the reference headphones (TDH39).

Through a 6cc coupler, a sound level meter was used to measure the peak power (in A-weighted dB, dBA) outputs from the standard audiometer and the tablet for each word at 20-, 30-, and 40-dB HL settings. The calibration coefficient calculated at each measuring point was essentially an additional gain required that would make the tablet output the word with the same dBA peak when measured using the sound level meter. The Beyerdynamic DT 770 PRO and the generic headphones with earmuffs were calibrated using the same procedure as the TDH39 headphones.

### Ethical Considerations

The study was approved by the Khon Kaen University Ethical Committee for Human Research (HE631548) and was registered in the Thai Clinical Trials Registry (TCTR20201229002). Written informed consent was obtained from all participants and participants were given the option to opt out. All data were deidentified. Compensation was provided for transportation and participation in the study.

### Statistical Analysis

#### Sample Size

The sample size was calculated from κ estimation with an expected κ value of 0.5±0.3. A power of 95% and a significance level of .05 were used. The ideal sample size was calculated to be 44, with a 20% dropout rate.

#### Data Analysis

Statistical analysis was conducted using STATA (StataCorp LLC). The agreement coefficiency, sensitivity, and specificity of the PASS-Pro app were determined. The McNemer test was used to compare the dichotomous data of the headphones with the reference headphones. A paired *t* test was used to compare average test duration of the app with conventional audiometry. For all tests, a *P* value of <.05 was considered to be statistically significant.

## Results

A total of 44 children participated in this study, comprising 19 (43%) female and 25 (57%) male participants. No participants dropped out of the study. Using standard conditioned play audiometry, we found that 31 (70%) children had normal hearing in both ears, 9 (21%) had mild or moderate unilateral hearing loss, and 4 (9%) had mild or moderate bilateral hearing loss. An otoscopic examination was performed on all children. Of the 44 children, 4 (45%) had a normal external ear canal and tympanic membrane and 5 (55%) had impacted cerumen.

**Figure 3.**
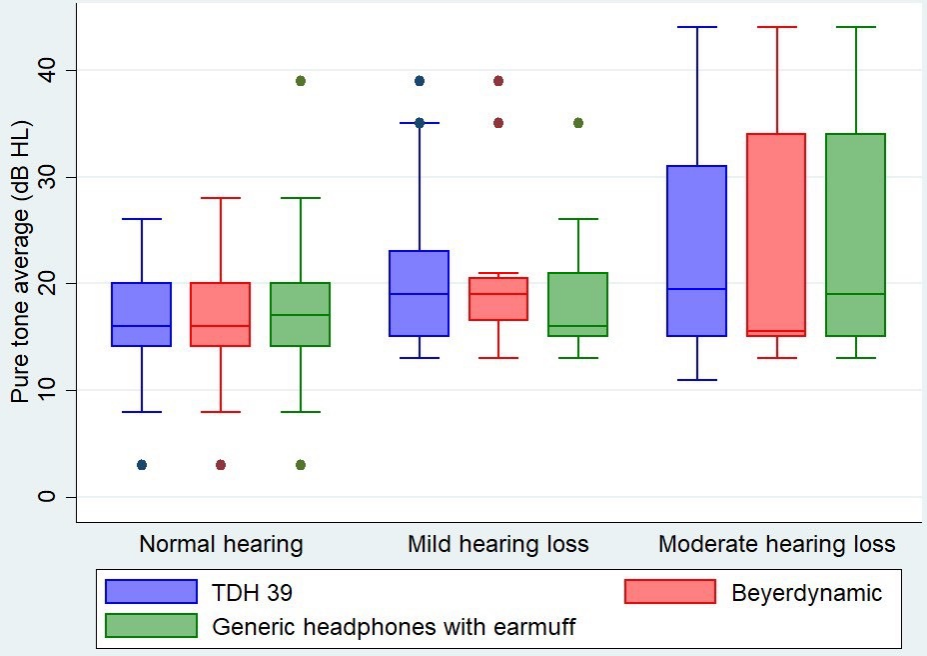
Pure tone average versus PASS-Pro’s (Preschool Audiometry Screening System–Pro) hearing screening levels. dB HL: dB hearing level.

The agreement between all headphones and standard conditioned play audiometry ranged from 69.52% to 94.32%, indicating good agreement between these tools. For mild hearing loss, the κ coefficients between the PASS-Pro app using TDH39 headphones, Beyerdynamic DT 770 PRO headphones, and generic earmuff headphones versus standard conditioned play audiometry were 0.195, 0.290, and 0.261 (*P*=.02, *P*=.002, and *P*=.004), respectively. For moderate hearing loss, the κ values between TDH39 headphones, Beyerdynamic DT 770 PRO headphones, and generic earmuff headphones versus standard conditioned play audiometry were 0.206, 0.272, and 0.235 (all *P*s=.001), respectively. The κ statistic indicated fair agreement between the headphones and audiometry (Table 2 and Figure 3).

**Table 2.** Agreement between the PASS-Pro (Preschool Audiometry Screening System–Pro) app and standard conditioned play audiometry (N=88).

Headphone type and hearing level		Agreement (%)	κ coefficient	*P* value^a^
**TDH39 headphones**				
	Normal hearing (n=69)	73.86	0.181	.04^b^
	Mild hearing loss (n=11)	83.06	0.195	.02^b^
	Moderate hearing loss (n=8)	92.05	0.206	.001^b^
**Beyerdynamic DT 770 PRO headphones**				
	Normal hearing (n=70)	69.52	0.068	.26
	Mild hearing loss (n=12)	87.19	0.290	.002^b^
	Moderate hearing loss (n=6)	94.32	0.272	.001^b^
**Generic headphones**				
	Normal hearing (n=62)	70.45	0.201	.02^b^
	Mild hearing loss (n=19)	89.77	0.261	.004^b^
	Moderate hearing loss (n=7)	91.09	0.235	.001^b^

a κ statistic.

b Indicates statistical significance.

The sensitivity of all headphones in detecting mild hearing loss was 50% and specificity was more than 88% whereas the sensitivity of all headphones in detecting moderate hearing loss was 100% and specificity was more than 92% (Table 3).

**Table 3.** Sensitivity and specificity of PASS-Pro (Preschool Audiometry Screening System–Pro) app (N=88).

Headphone type and hearing level		Sensitivity (95% CI)	Specificity (95% CI)	*P* value^a^
**TDH39 headphones**				
	Normal hearing (n=69)	37.5 (15.2-64.6)	81.9 (71.1-90)	—^b^
	Mild hearing loss (n=11)	50 (6.76-93.2)	88.1 (79.2-94.1)	—
	Moderate hearing loss (n=8)	100 (2.5-100)	92 (84.1-96.7)	—
**Beyerdynamic DT 770 PRO headphones**				
	Normal hearing (n=70)	25 (7.27-52.4)	81.9 (71.1-90)	.56
	Mild hearing loss (n=12)	50 (6.76-93.2)	92.9 (85.1-97.3)	.21
	Moderate hearing loss (n=6)	100 (2.5-100)	94.3 (87.1-98.1)	.45
**Generic headphones**				
	Normal hearing (n=62)	50 (24.7-75.3)	75 (63.4-84.5)	.12
	Mild hearing loss (n=19)	50 (6.76-93.2)	91.7 (83.6-96.6)	.37
	Moderate hearing loss (n=7)	100 (2.5-100)	93.1 (85.6-97.4)	.66

a McNemar test; comparison with reference headphones (TDH39).

b Not applicable.

The average test duration was 579.8 seconds (range 421-1152 seconds) for conditioned play audiometry, 91.27 seconds (range 58-150 seconds) for the PASS-Pro app with TDH39 headphones, 80.39 seconds (range 46-113 seconds) for the PASS-Pro app with Beyerdynamic DT 770 PRO headphones, and 84.39 seconds (range 50-130 seconds) for the PASS-Pro app with generic headphones. The PASS-Pro app used significantly less time than conditioned play audiometry (*P*<.001).

## Discussion

In this study, we found that the agreement between audiometry (the gold standard) and all headphones ranged from 69.52% to 94.32%. The κ statistic found a statistically significant correlation coefficient (all *P*s<.05) for all headphones. The sensitivity to detect mild hearing loss was 50% for all headphones while the specificity was more than 80%. Moreover, the sensitivity to detect moderate hearing loss was 100% for all headphones while the specificity was more than 90%. It can be inferred that generic headphones can be used for hearing screening.

Hearing loss among children is an important problem around the world. It significantly affects speech development, language learning skills, the thought process, communication skills, and social skills. Use of hearing screenings for early diagnosis and proper management may enhance quality of life [10,12,13]. However, in low- or middle-income countries, hearing screening tools are difficult to access [14]. There is a need to produce novel screening tools that are user-friendly, inexpensive, accessible, and practical for areas with limited resources to facilitate hearing screening.

A number of studies on hearing screening apps have been conducted, including Audioscope [15], HearCheck [9], SHOEBOX [16], and Tablet Hearing Game Screen [17]. Their sensitivity and specificity ranged from 80% to 91% and 80% to 100%, respectively.

In our previous study [11], the PASS app used with standard TDH39 headphones had a sensitivity and specificity of 76.67 and 95.83, respectively, for detecting mild hearing loss. In this study, we compared the diagnosis value of 3 different headphones. We found that the sensitivity for all headphones was 50% and specificity was more than 80% for mild hearing loss. For for moderate hearing loss, sensitivity for all headphones was 100% and specificity was more than 90%.

The discrepancy between the sensitivity results of 76.67% (95% CI 59.07%-88.21%) from our previous study versus 50% (95% CI 6.76%-93.2%) in this study may have occurred by chance. Our previous study of 122 children aimed to evaluate the sensitivity of the PASS app for hearing screening. However, the current study’s main objective was not to evaluate the sensitivity and sensitivity but to evaluate the agreement between the headphones and standard audiometry.

Our results agreed with those of other hearing screening apps that used various types of headphones. For example, Audiometer used earphones or earbuds [14], SHOEBOX Audiometry used earbuds [16], and the uHear and uHearing Test used 3 types of headphones (earbud headphones, supra-aural headphones, and circumaural headphones) [18]. All exhibited high sensitivity and specificity. Table 4 presents the sensitivity and specificity of current hearing screening apps.

**Table 4.** Comparison of the sensitivity and specificity of current hearing screening apps.

Measure	PASS-Pro^a^ (various headphones)	HearCheck (Ukoumunne et al [9])	PASS^b^ (Yimtae et al [11])	Tablet based (Xiao et al [17])
Sensitivity	50-100	89	76.67	91
Specificity	88.1-93.1	86.5	95.83	73.59

a PASS-Pro: Preschool Audiometry Screening System–Pro.

b PASS: Preschool Audiometry Screening System.

Although the agreement between audiometry (the gold standard) and all headphones was high, the κ correlation coefficient between these tools indicated fair agreement. This can be explained by Cohen’s Kappa Paradox, which is usually found in sensitivity studies. The effects of the paradox arise when participants tend to be classified into one of the possible outcomes. This is either due to the nature of the outcome itself and its high prevalence or because at least one of the evaluators tends to assign more frequently to a specific outcome (ie, the normal hearing group) [19].

Due to the limited number of participants with hearing loss in this study, increasing the sample size in future studies should provide more accurate results. The PASS-Pro app provides only spondee words; the next software update should allow the app to produce pure tone sound for better agreement with standard audiometry.

The standard reference audiometric speaker is typically supplied in the form of headphones. The design of the headphones allows it to be soundproof, suitable for high-frequency audiometry and high passive ambient noise attenuation. However, the headphones are large, heavy, and expensive whereas other types of devices such as earbuds and earphones are more portable and lightweight. Using earbuds or earphones for hearing screening may decrease the sensitivity of the system. However, to our knowledge, no study has been conducted to quantify this sensitivity difference yet.

For future research, researchers should study commercially available portable earphones instead of generic headphones with earmuffs as the former can be directly plugged into the ear canal, are low cost, and are widely available. In this study, we limited the hearing screening test to quiet hospital rooms; therefore, the next study will be tested in a quiet community setting such as a home or school to obtain a larger sample size and emulate a real-world environment.

In conclusion, the combination of the PASS-Pro app and generic headphones is a reliable method for conducting hearing screening tests in preschool children, offering both high sensitivity and specificity.

## Abbreviations

dB HL: dB hearing level
dBA: A-weighted dB
PASS: Preschool Audiometry Screening System
PASS-Pro: Preschool Audiometry Screening System–Pro
